# Long-Term Survival Associated with Direct Oral Feeding Following Minimally Invasive Esophagectomy: Results from a Randomized Controlled Trial (NUTRIENT II)

**DOI:** 10.3390/cancers15194856

**Published:** 2023-10-05

**Authors:** Tessa C. M. Geraedts, Teus J. Weijs, Gijs H. K. Berkelmans, Laura F. C. Fransen, Ewout A. Kouwenhoven, Marc J. van Det, Magnus Nilsson, Sjoerd M. Lagarde, Richard van Hillegersberg, Sheraz R. Markar, Grard A. P. Nieuwenhuijzen, Misha D. P. Luyer

**Affiliations:** 1 Department of Surgery, Catharina Hospital, 5623 EJ Eindhoven, The Netherlands; tessa.geraedts@catharinaziekenhuis.nl (T.C.M.G.); teus.weijs@catharinaziekenhuis.nl (T.J.W.); grard.nieuwenhuijzen@catharinaziekenhuis.nl (G.A.P.N.); 2 Department of Surgery, ZGT Hospital Group Twente, 7609 PP Almelo, The Netherlands; e.kouwenhoven@zgt.nl (E.A.K.); m.vandet@zgt.nl (M.J.v.D.); 3 Division of Surgery, Department of Clinical Science, Intervention and Technology (CLINTEC), Karolinska Institutet, 141-86 Stockholm, Sweden; magnus.nilsson@ki.se; 4 Department of Upper Abdominal Diseases, Karolinska University Hospital, 171-77 Stockholm, Sweden; 5 Department of Surgery, Eramus Medical Center, 3015 CN Rotterdam, The Netherlands; s.lagarde@erasmusmc.nl; 6 Department of Surgery, University Medical Center Utrecht, 3584 CX Utrecht, The Netherlands; r.vanhillegersberg@umcutrecht.nl; 7 Nuffield Department of Surgery, University of Oxford, Oxford OX3 9DU, UK; sheraz.markar@nds.ox.ac.uk

**Keywords:** early oral feeding, long-term outcomes, survival, minimally invasive esophagectomy

## Abstract

**Simple Summary:**

The direct start of oral intake after surgery improves short-term outcomes in patients undergoing minimally invasive esophagectomy. Based on recent insights, improvements in short-term outcomes may also lead to additional benefits in the long term. The current study investigated the overall survival and disease-free survival in patients receiving direct versus delayed oral feeding after minimally invasive esophagectomy in a randomized controlled cohort (NUTRIENT II trial). The current study showed that patients in the direct oral feeding group had improved 3-year overall survival and 5-year disease-free survival compared to standard care. These findings are unexpected and may provide a new target to improve long-term outcomes in this patient group.

**Abstract:**

Advancements in perioperative care have improved postoperative morbidity and recovery after esophagectomy. The direct start of oral intake can also enhance short-term outcomes following minimally invasive Ivor Lewis esophagectomy (MIE-IL). Subsequently, short-term outcomes may affect long-term survival. This planned sub-study of the NUTRIENT II trial, a multicenter randomized controlled trial, investigated the long-term survival of direct versus delayed oral feeding following MIE-IL. The outcomes included 3- and 5-year overall survival (OS) and disease-free survival (DFS), and the influence of complications and caloric intake on OS. After excluding cases of 90-day mortality, 145 participants were analyzed. Of these, 63 patients (43.4%) received direct oral feeding. At 3 years, OS was significantly better in the direct oral feeding group (*p* = 0.027), but not at 5 years (*p* = 0.115). Moreover, 5-year DFS was significantly better in the direct oral feeding group (*p* = 0.047) and a trend towards improved DFS was shown at 3 years (*p* = 0.079). Postoperative complications and caloric intake on day 5 did not impact OS. The results of this study show a tendency of improved 3-year OS and 5-year DFS, suggesting a potential long-term survival benefit in patients receiving direct oral feeding after esophagectomy. However, the findings should be further explored in larger future trials.

## 1. Introduction

Esophagectomy with or without neoadjuvant therapy is the cornerstone of curative treatment for esophageal cancer [1,2]. However, an esophagectomy is a complex surgical procedure that is associated with a notable complication rate [3,4]. Advancements in perioperative care, such as the introduction of minimally invasive surgery and enhanced recovery after surgery (ERAS) programs, have significantly reduced postoperative morbidity and improved recovery [5,6,7]. 

Our previous findings have indicated that further optimization of ERAS can be achieved by initiating oral intake directly after minimally invasive esophagectomy (MIE) [8,9]. In this multicenter randomized controlled trial, we demonstrated that the direct initiation of oral intake following minimally invasive esophagectomy is feasible and safe. The occurrence of anastomotic leakage and pulmonary complications was similar to the delayed start of intake [8]. Subsequently, a larger single-center cohort showed that the direct start of oral intake reduced the length of hospital stay and overall complication rates when compared to a delayed start [9].

Interestingly, a reduction in short-term complications not only benefits short-term outcomes, but is also associated with improved long-term survival [10,11,12]. Overall complications, anastomotic leakage, and cardiopulmonary complications after MIE were linked to decreased long-term survival. Patients without anastomotic leakage experienced an absolute 5-year survival benefit of 13.2% [10,11]. Improving outcomes in the short-term may therefore substantially impact the long-term survival of patients undergoing an esophagectomy.

In this planned sub-study of the randomized controlled NUTRIENT II trial, we investigated the influence of direct oral intake versus delayed oral intake on long-term survival.

## 2. Materials and Methods

### 2.1. Study Design and Participants 

Patients who participated in the NUTRIENT II trial were included in this follow-up study. Patients who died within 90 days of surgery were excluded to solely focus on long-term outcomes after esophagectomy [11,13,14]. 

The NUTRIENT II trial was a prospective, international, multicenter, open-label, randomized controlled trial executed in three hospitals (Catharina Hospital (Eindhoven, The Netherlands), Hospital Group Twente (Almelo, The Netherlands), and Karolinska University Hospital (Stockholm, Sweden)), and included patients aged 18 years or above who underwent a minimally invasive Ivor Lewis esophagectomy (MIE-IL) between 1 October 2015 and 14 May 2018. The participants were randomized prior to surgery to receive either direct oral feeding (intervention group) or delayed oral feeding (control group) following esophagectomy. A detailed description of the methodology was published previously [8,15]. The NUTRIENT II trial was registered at ClinicalTrials.gov with registration number NCT02378948 and at the Dutch trial registry with registration number NTR4972.

### 2.2. Surgical Procedure

All patients underwent an MIE-IL with a two-field lymphadenectomy. Intrathoracic anastomoses were performed side-to-side stapled or end-to-end hand sewn robotically. A feeding jejunostomy was routinely placed in all patients and was only used in the control group or in the occurrence of complications that prohibited oral intake in the intervention group. After surgery, the patients stayed in the ICU or a specialized postoperative ward unit for one night before returning to a regular surgical ward as part of the standard postoperative protocol. Standardized postoperative care for all patients also included early mobilization, no nasogastric tube placement, and optimal pain management. 

### 2.3. Nutritional Intervention

In patients allocated to the intervention group, oral intake (pureed and liquid food) was started directly after surgery. The patients were allowed to drink sips of water up to 250 cc on the day of surgery, and 500 cc of oral intake (i.e., soups, pureed food, and nutritional drinks) was allowed on postoperative day (POD) 1. The intake was gradually increased to a maximum of 1500 cc on POD 5. From POD 15 onwards, the patients could eat solid foods without any restrictions. 

The patients in the control group (delayed oral feeding) had a delay in oral intake and were only allowed to drink sips of water up to 250 cc in the days following surgery. Additionally, they received nutrition through the jejunostomy. From POD 5 onwards, the patients were allowed to gradually increase their oral intake similarly to the intervention group. The intake of solid food without restrictions was allowed from POD 15. The complete nutritional protocol of both treatment arms was disclosed in earlier work [8]. 

Tube feeding via jejunostomy was initiated in patients for whom complications prohibited oral intake or in patients in the intervention group with an intake lower than 50% of their calculated caloric need on POD 5. A routine chest X-ray was performed daily, and when gastric conduit dilatation was visible or clinical signs of gastroparesis emerged, a nasogastric tube was (endoscopically) inserted.

### 2.4. Follow-Up

Routine follow-up visits were scheduled postoperatively according to the local protocol within each hospital. On indication, visits were scheduled earlier. In general, no routine radiologic investigations were carried out and diagnostic assessments were primarily guided by the symptoms of the patient. After five years, no routine follow-up visits were planned and visits were only planned on indication. 

### 2.5. Outcome and Definitions

Comparisons were made between the delayed and direct oral feeding groups. The primary outcome was overall survival, defined as all deaths occurring within 3 and 5 years from the time of surgery from any cause. The secondary outcome was disease-free survival, defined as locoregional recurrence or metastatic disease diagnosed within 3 and 5 years from the time of surgery that was related to the primary esophageal cancer. Other outcomes included the impact of complications and caloric intake on 3- and 5-year overall survival. 

### 2.6. Data Collection and Statistical Analysis 

Patient characteristics and postoperative data until 90 days after surgery were prospectively collected. Recurrence and vital status data were prospectively registered and retrospectively collected from the electronic patient dossier. If not up to date, vital status data were obtained by assessing the municipal administrative database, which contains information about all (former) inhabitants of a country. 

Categorical data were assessed using the χ^2^ test or Fisher’s exact test and presented as absolute values with a corresponding percentage. After an assessment of normality, continuous variables were analyzed using the T-test or Mann–Whitney U and presented as the median with interquartile range (IQR). Analyses were carried out according to the intention-to-treat principle. A two-tailed *p*-value of <0.05 was considered statistically significant. Statistical analyses were performed using Statistical Software for the Social Sciences (SPSS) version 29 (IBM Software Group). Figures were made with Prism 8.2 (GraphPad Software, La Jolla, CA, USA).

The 5-year overall and disease-free survival in the delayed and direct oral feeding groups were estimated using the Kaplan–Meier method and compared with the log-rank test. Time to event was calculated from the date of surgery to the date of event (death/recurrence) or last follow-up (censor). Patients were censored if they were alive 5 years after surgery. The overall survival and disease-free survival rates at 3 years were compared between the delayed and direct oral feeding group using the χ^2^ test. 

Further analyses were conducted to assess the impact of additional characteristics on 3-year and 5-year overall survival within this cohort. To evaluate the influence of postoperative complications on overall survival, analyses were performed separately for patients with and without complications occurring within 30 days of surgery. Also, the impact of the severity of complications according to the CD classification was investigated (i.e., no complication, CDI-II, CD > III). Moreover, the impact of caloric intake on survival outcomes was examined. The percentage of caloric need was calculated (i.e., ratio of caloric intake/calculated caloric need) on POD5 and divided into quartiles. The overall survival of these quartiles was compared. The 5-year overall survival between groups was estimated using the Kaplan–Meier method and compared with the log-rank test. The 3-year survival was compared between groups using the χ^2^ test.

## 3. Results

### 3.1. Study Population

In total, 145 of 148 patients who participated in the NUTRIENT II trial were included in this follow-up study. Three patients were excluded because of death within 90 days of surgery. Of the included patients, 63 (43.4%) received direct oral feeding after esophagectomy.

### 3.2. Baseline Characteristics and Postoperative Outcomes

The baseline characteristics are shown in Table 1. The patients were predominantly male (86.0%) and had a median age of 65 years (IQR 60–70). Comorbidities were present in 68.2% of patients, with no differences in the distribution of comorbidities between groups. The tumor characteristics were similar in both groups and nearly all patients received neoadjuvant treatment (92.2%). The groups were comparable in terms of alcohol consumption and smoking status Data on perioperative and postoperative complications were previously published [8]. Perioperative complications did not differ between groups. The overall incidence of postoperative complications was 76.7% in all patients. Pulmonary complications were most prevalent in both groups. Chyle leakage was more frequently observed in the delayed oral feeding group when compared to the direct oral feeding group (10.6% vs. 1.6%, *p* = 0.034). Other postoperative complications, including pneumonia, anastomotic leakage, postoperative ileus, conduit necrosis/fistula, and jejunostomy complications, were equally distributed between both groups. Also, the severity of complications according to the Clavien–Dindo (CD) classification was comparable. 

### 3.3. Primary Outcomes

**Figure 1:**
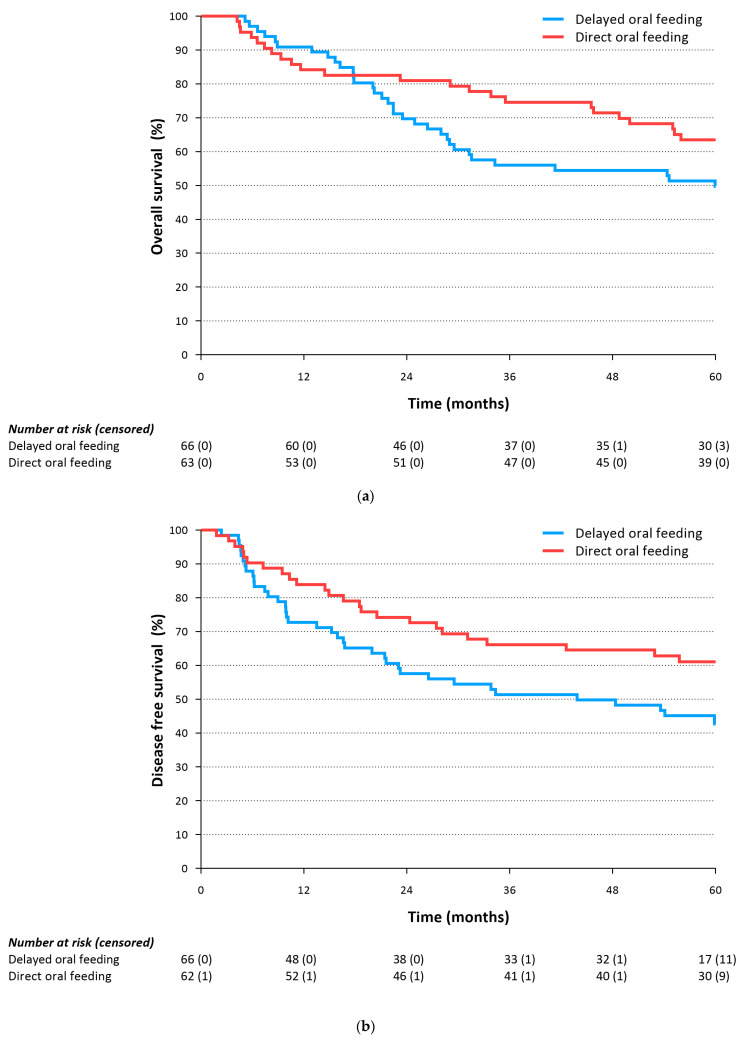
(**a**) Overall survival of the delayed oral feeding and direct oral feeding group (log-rank: *p* = 0.115). (**b**) Disease-free survival of the delayed oral feeding and direct oral feeding groups (log-rank: *p* = 0.047).

After five years of follow-up, 46.2% of patients were alive in the delayed oral feeding group compared to 61.9% in the direct oral feeding group (*p* = 0.115, Figure 1a). The median overall survival was 60.0 months (IQR NE (not estimable)-21.8) in the delayed oral feeding group and it was not reached (IQR NE–35.5) for patients in the direct oral feeding group. At 3 years follow-up, the number of patients alive in the direct oral feeding group was significantly higher than in the delayed oral feeding group (74.6% vs. 56.1%, *p* = 0.027). All patients in this cohort died as a result of metastatic disease from the primary esophageal cancer, with the exception of one patient in each group who died due to non-cancer related causes (cardiac disease, hospital-acquired pneumonia).

### 3.4. Secondary Outcomes

For disease-free survival, the Kaplan–Meier curves showed an early separation between groups favoring the direct oral feeding group that continued over time; however, this did not reach statistical significance at 3 and 5 years. Disease-free survival at 5-year follow-up was 33.3% versus 52.9% (*p* = 0.047, Figure 1b) for patients allocated to the delayed and direct oral feeding groups, respectively. The median disease-free survival in the delayed oral feeding group was 43.9 months (IQR NE-10.0), and for patients in the direct oral feeding group, the median disease-free survival was not reached (IQR NE-20.5) at 5-year follow-up. After 3 years, 50.7% of the patients in the delayed oral feeding group had no disease recurrence versus 66.1% in the direct oral feeding group (*p* = 0.065). In the direct oral feeding group, recurrence data were missing for one person and three patients were lost to follow-up (between 48 and 53 months after surgery).

### 3.5. Impact of Complications on Overall Survival

**Figure 2:**
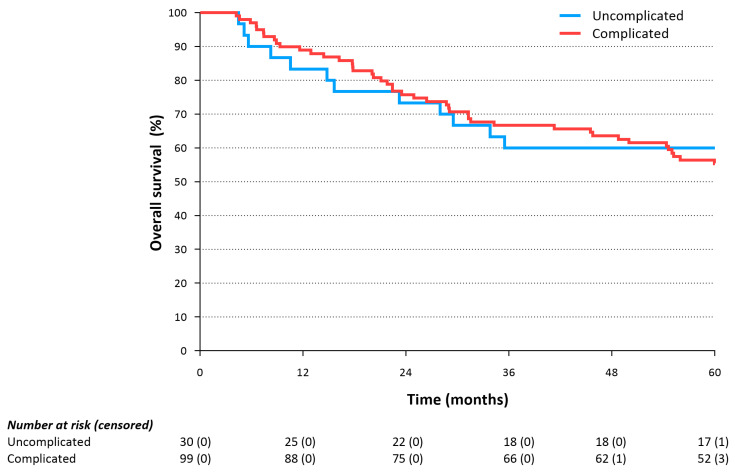
Overall survival in patients with an uncomplicated and complicated postoperative course following esophagectomy (log-rank: *p* = 0.843).

The characteristics of patients with or without complications were comparable at baseline. In this cohort, the occurrence of postoperative complications did not seem to influence 3- (*p* = 0.502) and 5-year overall survival (*p* = 0.843, Figure 2). Moreover, the grade of complications according to the Clavien–Dindo classification did not affect the 3- and 5-year overall or disease-free survival (*p* = 0.956 and *p* = 0.691).

### 3.6. Impact of Caloric Intake on Overall Survival

**Figure 3:**
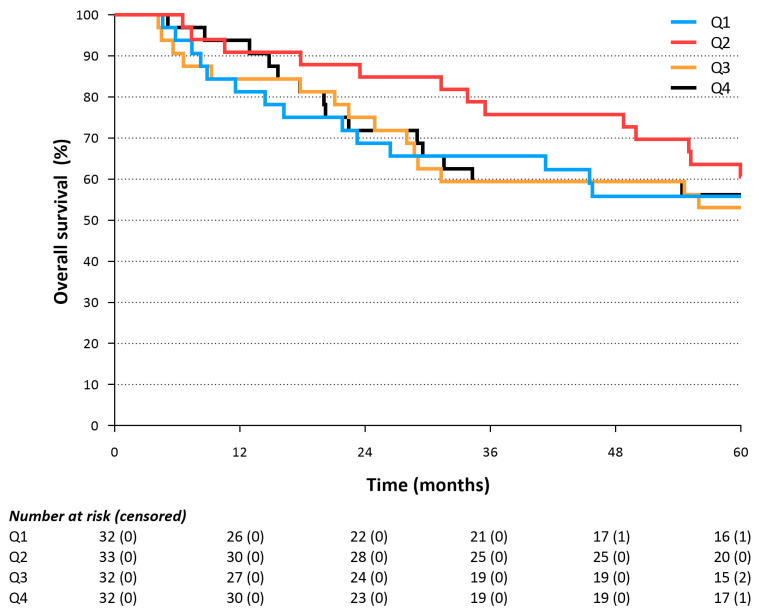
Overall survival of the quartiles of the percentage of caloric need (i.e., ratio of caloric intake/calculated caloric need) on postoperative day 5 (log-rank: *p* = 0.972).

The median caloric intake on POD 5 was 1220 kcal (IQR 900–1430) in the early oral intake group versus 1968 kcal (IQR 1678–2410, *p* < 0.001) in the delayed oral feeding group. The corresponding median percentage of caloric need was 96.0% (IQR 74.6–112.2%) and 55.9% (44.3–68.0%, *p* < 0.001) in the delayed and direct oral feeding group, respectively. After segmenting the percentage of caloric need into quartiles and subsequently comparing them in relation to overall survival, no differences among the quartiles were observed at 3 and 5 years (*p* = 0.461 and *p* = 0.972, Figure 3).

## 4. Discussion

This long-term follow-up study of the previously published randomized NUTRIENT II trial investigated the impact of direct oral feeding compared to delayed oral feeding on survival after MIE. The results suggest that direct oral feeding may potentially affect the overall survival of patients undergoing esophagectomy. To our knowledge, this is the first study investigating the effects of the early initiation of oral feeding after MIE on long-term survival in a randomized controlled cohort. 

The benefits of directly starting oral intake following abdominal surgery have been shown in multiple randomized controlled trials and are a cornerstone of contemporary ERAS protocols. Direct oral feeding reduces metabolic and hormonal stress, improves recovery, shortens the length of hospital stay, and enhances patient satisfaction [16,17,18,19,20,21,22,23,24]. Outcomes from previous trials performed by our group indicate that direct oral intake enhances postoperative recovery after esophagectomy [8,9,25]. Since improved postoperative recovery is associated with better long-term overall survival, direct oral intake might positively influence long-term survival [10,11,12,14]. The results from this sub-study are in line with this hypothesis, although after 5 years of follow-up, no statistical difference was observed between the groups. 

Additionally, recurrence data show improved 5-year disease-free survival and indicate a trend towards improved disease-free survival at 3 years favoring the direct oral intake group, though at 3 years, statistical significance was not reached. Berkelmans et al. showed no differences between direct and delayed oral intake in overall and disease-free survival after 24 months in esophagectomy patients [26]. However, this was a retrospective cohort study with a short follow-up period, which is different from the current randomized controlled setup with an adequate follow-up period. 

Importantly, the potential association between direct oral intake and survival observed in our analysis is surprising and not fully understood. Hence, we conducted additional analyses to explore the predictors of survival within this cohort. The occurrence of complications is one of the factors for which a relationship with long term survival has been previously suggested [10,11,12,14,27,28,29]. In this study, no differences in postoperative complications were found between groups, apart from a higher incidence of chyle leakage in the delayed oral intake group. The few studies that have investigated the influence of chyle leakage on long-term survival present ambivalent results. The study by Hagens et al. shows that chyle leakage is an independent predictor for survival after esophagectomy [30], whereas the study by Milito et al. shows that the presence of chyle leakage does not negatively affect long-term outcomes [31]. In contrast with the previous literature, the occurrence and severity of postoperative complications did not affect long-term survival in this study. This discrepancy might be attributable to the relatively small study size, comprising only 30 patients (23.3%) in the uncomplicated course group. 

Regarding caloric intake, the initial NUTRIENT II trial showed that patients in the direct oral feeding group had a significantly lower intake on POD2, 5, and 14 when compared to the delayed oral feeding group, without leading to BMI differences between groups. This was to be expected as oral intake is reduced directly after esophagectomy, and an intake of at least 50% on POD5 was accepted in the direct oral feeding group. In the control group, the patients received increasing volumes of tube feeding [8]. Variation in caloric intake could potentially impact long-term survival by interacting with tumor metabolism. Pre-clinical research suggests that caloric restriction—reducing the intake by approximately 20–40%—might hold the potential to regulate tumor growth and progression [32,33,34,35,36]. In turn, this could influence cancer recurrence rates. Some in-human evidence of the potential benefits of caloric reduction for cancer is available. A small pilot study in patients with newly diagnosed prostate cancer showed that a 6-week calorie-restricted diet (i.e., 30% reduction) resulted in changes in serum proteins that are potentially related to prognosis [37]. Moreover, a case–control study demonstrated that the consumption of fewer calories (i.e., 20% reduction) was associated with a decreased risk of breast cancer in premenopausal women [38]. But up to now, no prospective studies have been executed that confirm the potential protective effect of caloric restriction in cancer patients. Overall, the in-human evidence for the role of caloric restriction on cancer growth and progression is limited and the best application of caloric restriction in cancer patients is uncertain.

Next, the preoperative feeding status of patients could have played a role in the current outcomes. As esophageal cancer affects the functionality of the esophagus and subsequent nutritional intake, the preoperative nutritional status is often compromised in this patient group [39,40]. It is possible that the patients, in whom intake was already restricted before surgery, experienced a more profound effect from the nutritional intervention (i.e., lower caloric intake) in the long term. Unfortunately, no data on preoperative intake were present for the patients included in NUTRIENT II in order to further explore this hypothesis. 

Finally, the proportion of oral and (par)enteral intake could have contributed to the outcomes. The study by Okada et al. showed that postoperative nutrition, mostly based on enteral intake, negatively influenced overall survival when compared to oral intake as a more physiological route [41], underlining the importance of prioritizing oral nutrition over parenteral or enteral nutrition [40,42,43]. In the delayed oral feeding group, the oral intake was restarted five days later than in the direct oral feeding group. Furthermore, from the cohort study by Berkelmans et al., it can be concluded that significantly more nutritional interventions (i.e., enteral or parenteral reinterventions) were needed for the patients receiving delayed oral feeding when compared to direct oral feeding [26]. This may indicate that patients who receive delayed oral feeding have a more unfavorable ratio of oral and (par)enteral nutrition, which might affect outcomes on the long term. Yet, based on the available data from the NURIENT II trial and the current long-term follow-up study, an underlying nutritional cause for the observed effect cannot be completely established.

The current study holds an important strength as it builds upon the multicenter randomized controlled NUTRIENT II trial. The patients in this trial were not selected based on advantageous characteristics, making the results comparable with daily clinical practice. Additionally, most data were prospectively collected as part of the standard follow-up. However, this study also has some limitations. Recurrence data were retrospectively collected from the electronic patient dossier and, therefore, some recurrence data are missing (*n* = 1) or incomplete (*n* = 3). Also, the relatively small sample size of the NUTRIENT II cohort might have influenced the statistical power to detect certain long-term associations. Although this is a planned sub-study of the NUTRIENT II trial, the initial study was powered for functional recovery and it might well be that it was underpowered for accurately assessing long-term survival outcomes. 

## 5. Conclusions

The outcomes of the current follow-up study from the randomized controlled NUTRIENT II trial show a trend towards improved 3-year overall survival and 5-year disease-free survival in patients receiving direct oral feeding following MIE-IL when compared to delayed oral feeding. These findings suggest a potential survival benefit associated with direct oral feeding. However, additional research is needed to substantiate a potential relation between this intervention and long-term survival. 

## Figures and Tables

**Table 1:** Comparison of baseline characteristics between the delayed and direct oral feeding groups.

	Delayed Oral Feeding *n* = 66	Direct Oral Feeding *n* = 63	*p*-Value *
	57 (86.4)	54 (85.7)	0.915
**Age at randomization, years**	65 [61–70]	66 [59–70]	0.853
**BMI at diagnosis, kg/m^2^**	26 [24–29]	26 [23–29]	0.441
**ASA score**			0.749
**I**	9 (13.6)	6 (9.5)	
**II**	42 (63.5)	43 (68.3)	
**III-IV**	15 (22.7)	14 (22.2)	
**Comorbidities**			
**Overall**	48 (72.7)	40 (63.5)	0.260
**Vascular**	21 (31.8)	20 (31.7)	0.993
**Cardiac**	17 (25.8)	8 (12.7)	0.061
**Pulmonary**	7 (10.6)	8 (12.7)	0.711
**Diabetes**	6 (9.1)	8 (12.7)	0.510
**Tumor location**			0.565
**Mid**	3 (4.5)	1 (1.6)	
**Distal**	46 (69.7)	43 (68.3)	
**GEJ**	17 (25.8)	19 (30.2)	
**Tumor histology**			0.549
**Adenocarcinoma**	54 (81.8)	54 (85.7)	
**Squamous-cell carcinoma**	12 (18.2)	9 (14.3)	
**Neoadjuvant treatment**	63 (95.5)	56 (88.9)	0.163
**Clinical tumor stage**			0.787
**I**	12 (18.5)	11 (17.5)	
**II**	20 (30.8)	23 (36.5)	
**III**	33 (50.8)	29 (46.0)	
**Lymph nodes harvested, number**	22 [17–27]	23 [18–30]	0.252
**Total positive lymph nodes, number**	0 [0–1]	0 [0–1]	0.775
**Radicality of resection**			
**R0: microscopic radical**	65 (98.5)	63 (100)	0.327
**R1: microscopic irradical**	1 (1.5)	0 (-)	
**Pathological T stage**			0.202
**T0**	11 (19.7)	20 (31.7)	
**T1**	18 (27.3)	15 (23.8)	
**T2**	10 (15.2)	10 (15.9)	
**T3**	27 (40.9)	18 (28.6)	
**Pathological N stage**			0.102
**N0**	47 (71.2)	41 (65.1)	
**N1**	7 (10.6)	15 (23.8)	
**N2-3**	12 (18.2)	7 (11.1)	

* *p*-values < 0.05 are considered statistically significant. *GEJ* indicates gastrointestinal junction. Values are presented as absolute numbers (%) or median [interquartile range].

## Data Availability

The data presented in this study are available on request from the corresponding author. The data are not publicly available as it contains information that could compromise the privacy of research participants.
